# Prognostic value of soluble ST2 in cardiac magnetic resonance defined myocardial infarction with non-obstructive coronary arteries

**DOI:** 10.3389/fcvm.2026.1917954

**Published:** 2026-09-15

**Authors:** Chenchen Cui, Donglin Liu, Mingchang Du, Peng Lu, Yu Yang, Lei Chen, Wensu Chen, Min Zhang

**Affiliations:** 1 Department of Neurology, The Affiliated Hospital of Xuzhou Medical University, Xuzhou, China; 2 Department of Cardiology, The Affiliated Hospital of Xuzhou Medical University, Xuzhou, China

**Keywords:** cardiac magnetic resonance, global longitudinal strain, major adverse cardiac events, MINOCA, soluble suppression of tumorigenicity 2

## Abstract

**Background:**

Cardiac magnetic resonance (CMR) plays an essential role in identifying the underlying etiology of myocardial infarction with non-obstructive coronary arteries (MINOCA). However, risk stratification in patients with CMR-confirmed MINOCA remains insufficiently defined. Soluble suppression of tumorigenicity 2 (sST2), a biomarker related to myocardial fibrosis and adverse cardiac remodeling, may provide additional prognostic information in this population. This study aimed to evaluate the prognostic value of sST2 in patients with CMR-confirmed MINOCA.

**Methods:**

This single-center retrospective study included patients suspected of MI who underwent coronary angiography and completed CMR during hospitalization. MACE included all-cause death, recurrent MI, stroke, heart failure, arrhythmia, and angina pectoris. MINOCA was defined by evidence of ischemia or infarction on CMR. sST2 was tested using an immunoassay kit according to the protocol during hospitalization.

**Results:**

A total of 97 patients with CMR-confirmed MINOCA were included. During a median follow-up of 24.2 months, 33 patients experienced MACE. Patients with MACE had significantly higher sST2 levels and worse CMR parameters, including lower left ventricular ejection fraction, greater late gadolinium enhancement extent, larger left ventricular end-systolic volume index, and impaired global longitudinal strain (GLS). Multivariable Cox regression analysis showed that sST2 (HR 1.010, 95% CI 1.003–1.017, *P* = 0.005) and GLS (HR 0.864, 95% CI 0.780–0.956, *P* = 0.005) were independently associated with MACE. Receiver operating characteristic analysis demonstrated moderate predictive performance for sST2, with an area under the curve of 0.696. Kaplan–Meier analysis showed that patients with sST2 ≥ 36.95 ng/mL had a significantly higher risk of MACE.

**Conclusions:**

Elevated sST2 is independently associated with adverse outcomes in patients with CMR-confirmed MINOCA. sST2 may serve as a practical biomarker for risk stratification in this population.

## Introduction

Myocardial infarction (MI) with non-obstructive coronary arteries (MINOCA) is one of the common types of MI and is characterized by less than 50% stenosis of major epicardial vessels on coronary angiography (CAG) (1). MINOCA is not a benign process. Long-term follow-up showed that MINOCA patients had a significantly increased risk of major adverse cardiovascular events (MACE) (2). Unfortunately, as a heterogeneous disease with multiple causes, there is still no unified and effective clinical management method for MINOCA (3). Investigating and identifying the specific underlying cause of MINOCA is critical for patients and physicians. The 2019 American Heart Association (AHA) statement proposed that once Takotsubo syndrome (TS), myocarditis, and non-ischemic cardiomyopathy are diagnosed, patients are no longer considered to have MINOCA because they do not meet the fourth universal definition of MI (1, 4). Cardiovascular magnetic resonance (CMR) allows characterization of myocardial tissue, thereby distinguishing various potential etiologies, with reported diagnostic yields ranging from 33% to 92% (5–8). In addition, previous studies have shown that patients with cardiomyopathy and MI diagnosed by CMR have a poor prognosis, and patients with normal CMR have the best prognosis (9, 10). In another study, Ananthakrishna et al. found that CMR diagnosis of MI was an independent predictor of MACE (11). For the management of patients with MINOCA, both American and European statements advocate the use of CMR to investigate the underlying etiologies (1, 12). However, prognostic stratification regarding CMR confirmed MINOCA is still lacking.

Myocardial fibrosis is a key link in the onset and progression of cardiovascular diseases, which has also been confirmed in MINOCA patients (13, 14). ST2 is a member of the interleukin (IL-1) receptor family, and there are two subtypes: transmembrane receptor (ST2L) and soluble receptor (sST2) (15). IL33/ST2L has been shown to inhibit myocardial fibrosis and exert a cardioprotective effect. In the case of cardiac injury, increased sST2 can interfere with the IL-33/ST2L signaling pathway by competitively binding to IL-33 and negate its cardioprotective effect (16, 17). As a marker of myocardial fibrosis, sST2 has been confirmed to be associated with left ventricular (LV) remodeling and increased risk of MACE after MI (15–17), and has been included in the 2013 AHA heart failure management guidelines (18). Recently, Gruson D et al. found that sST2 was the strongest predictor of cardiac death in a multivariate model including age, left ventricular ejection fraction (LVEF), and N-terminal pro-B-type natriuretic peptide (NT-proBNP), and estimated glomerular filtration rate (19).

However, the prognostic value of sST2 in MINOCA patients remains unclear. In addition, most current studies on the prognosis of MINOCA did not explicitly exclude patients with non-ischemic origin. This study used CMR to reclassify MINOCA patients and explore the characteristics of sST2 in different etiologies and the prognostic value of sST2 in patients with MINOCA.

## Methods

### Study population

This single-center retrospective study included patients suspected of MI who underwent CAG at the Affiliated Hospital of Xuzhou Medical University between June 2020 and November 2025. Inclusion criteria: Coronary artery stenosis of less than 50%; Peak high-sensitivity troponin T (hs-TnT) exceeding the 99th percentile; sST2 and CMR examination during hospitalization. Exclusion criteria: Poor CMR images; Diagnosed with TS, myocarditis, other non-ischemic cardiomyopathies, or normal by CMR; Loss of follow-up. The Institutional Review Board (IRB) of the Affiliated Hospital of Xuzhou Medical University approved this study protocol (NO. XYFY2026-KL154-01). The requirement for signed written consent was waived owing to the no risk to the patient in accordance with the relevant IRB regulatory guidelines. This research was conducted in accordance with the Declaration of Helsinki. In total, 97 patients were diagnosed with MINOCA by CMR (Figure 1). Baseline clinical data were collected for all patients by reviewing electronic medical records, which included age, sex, cardiovascular risk factors, and discharge medications. During hospitalization, blood samples were collected for standard analysis. The peak values for hsCRP, NT-proBNP, and hsTnT were recorded. sST2 was tested using an immunoassay kit (provided by Springbio, Guangzhou, China) according to the protocol. sST2 was measured during the index hospitalization. Laboratory personnel performing the sST2 assay were unaware of subsequent clinical outcomes. Exact intervals from symptom onset to sST2 sampling and coronary angiography were not consistently available because of the retrospective design.

**Figure 1:**
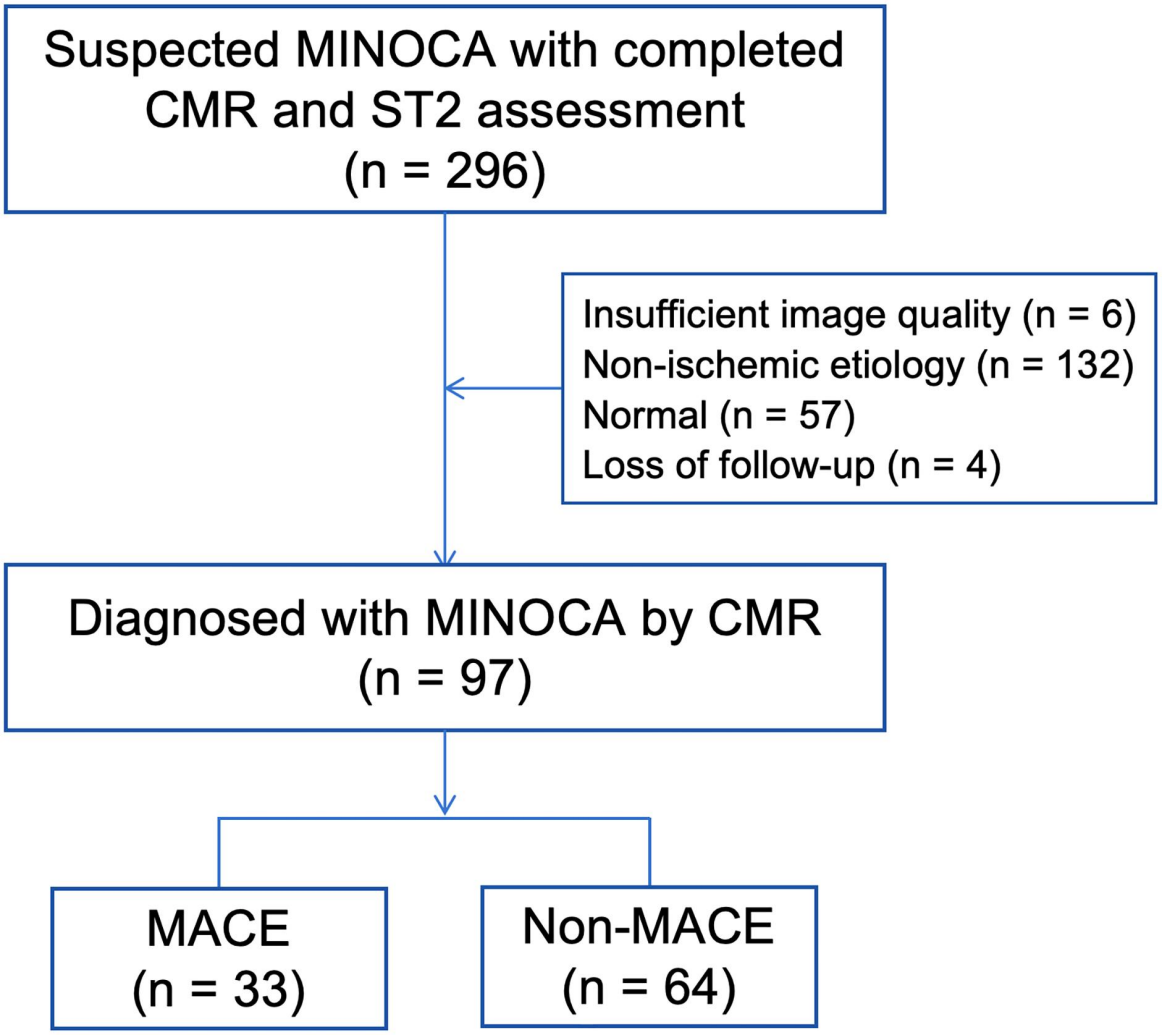
Study flowchart. CMR, cardiac magnetic resonance; sST2, soluble suppression of tumorigenicity 2; MINOCA, myocardial infarction with non-obstructive coronary arteries; TS, takotsubo syndrome; CAG, coronary angiography.

### CMR protocol and related parameters

As described in our previously published papers (20, 21), CMR was performed on 3.0T systems (Ingenia, Philips, The Netherlands). CMR was performed at a median of 3 days after admission. A balanced turbo field echo (BTFE) sequence was adopted. CMR images (cine imaging, late gadolinium enhancement, T2 and/or T1 imaging) were acquired. The CVI42 (cvi42® version 5.13.5, Circle Cardiovascular Imaging, Canada) was used for image analysis. CMR image analysis was performed independently by 2 experienced doctors. End-diastolic volume (EDV), end-systolic volume (ESV), and left ventricular ejection fraction (LVEF) were automatically obtained and corrected for body surface area (BSA). CMR-FT was used to obtain the LV global longitudinal strain (GLS). All strains were analyzed by taking absolute values. Intraobserver agreement (ICC, 0.959; 95% CI, 0.925–0.974) and interobserver agreement (ICC, 0.955; 95% CI, 0.931–0.976) were excellent for GLS in a randomly selected subset of 30 patients. Microvascular obstruction (MVO) was observed from the late gadolinium enhancement (LGE) images. LGE% was expressed as a percentage of the total left ventricular myocardial mass.

### CMR reclassifies

Combined with relevant definitions, reclassification was performed based on the presence of wall motion abnormalities on CMR, LGE images, T2-weighted imaging, and/or T1 imaging. All patients were classified as MINOCA, myocarditis, TS, cardiomyopathy (other non-ischemic cardiomyopathies, including dilated cardiomyopathy, hypertrophic cardiomyopathy, arrhythmogenic cardiomyopathy, etc.), and normal. The specific definitions were as follows. MINOCA was characterized by LGE or myocardial edema in the subendocardial or transmural region of the coronary territory (22). TS, Myocarditis and other non-ischemic cardiomyopathies were identified in accordance with relevant guidelines or consensus statements (23–25).

### Clinical outcomes and follow-up

Patients were followed up from the time of discharge through outpatient visits and/or telephone contact using a standardized questionnaire. MACE was defined as a composite of all-cause death, recurrent MI, stroke, hospitalization for heart failure, atrial fibrillation, and hospitalization for angina. Recurrent MI and heart failure was diagnosed according to current ESC guidelines (4, 26). Stroke was defined as neurological dysfunction and cerebrovascular injury caused by cerebral ischemia or cerebral hemorrhage (27). Atrial fibrillation referred to newly diagnosed AF during follow-up. Angina pectoris was defined as recurrent angina requiring rehospitalization. All patients lost to follow-up were confirmed deceased through the local official household registry. All clinical events were independently reviewed by two investigators. In cases of disagreement, a third experienced investigator adjudicated the event, and the final classification was determined by consensus.

### Statistical analysis

Statistical analysis was performed using SPSS 27.0 (IBM, Chicago, USA) and R (Lucent Technologies, New Jersey, USA). Continuous variables with a normal distribution were presented as mean ± standard deviation (SD) and analyzed using Student's *t*-test. Non-normally distributed continuous variables were reported as median (Q25, Q75) and analyzed with the Mann–Whitney *U*-test. Categorical variables were expressed as frequencies and percentages and analyzed using the chi-square test. Cox proportional hazards regression models were used to evaluate associations with MACE. Variables with a *P* < 0.10 in univariable Cox regression analyses were considered candidate covariates for the forward stepwise multivariable Cox model. Given the limited number of outcome events, model complexity was evaluated using the events-per-variable (EPV) ratio. To assess the robustness of the primary findings while limiting model dimensionality, two additional fixed parsimonious sensitivity Cox models were constructed without stepwise variable selection. Model 1 included sST2, age, and GLS, whereas Model 2 included sST2, age, and LGE. Age was included as an established clinical prognostic factor, while GLS and LGE were evaluated separately to account for functional and tissue-injury CMR characteristics, respectively. All covariates in the sensitivity models were entered simultaneously. To further account for clinically relevant confounders irrespective of univariable significance, additional fixed sensitivity Cox models were constructed for demographic/comorbidity factors, biomarkers/renal function, and discharge medications. All covariates were entered simultaneously without stepwise selection. Multicollinearity among candidate predictors was assessed using the variance inflation factor (VIF), with VIF <5 considered acceptable. No substantial multicollinearity was observed among the candidate predictors (VIF range, 1.05–1.44). The proportional hazards assumption for the Cox regression models was assessed using Schoenfeld residuals, with *P* > 0.05 indicating no evidence of violation. Restricted cubic splines (RCS) were used to investigate the dose-response relationship between sST2 and MACE. Conventional ROC analysis was used to explore the discrimination of sST2 and GLS for MACE. The incremental prognostic value of sST2 beyond conventional CMR parameters was evaluated by comparing a base Cox model including LVEF, GLS, and LGE with an expanded model additionally including sST2. Overall improvement in model fit was assessed using the likelihood ratio test (LRT) for the nested Cox models. In addition, continuous net reclassification improvement (NRI) and integrated discrimination improvement (IDI) were evaluated at a 24-month time horizon, which closely approximated the median follow-up duration of the cohort (24.2 months). Predicted 24-month MACE risks were derived from the Cox models, and right censoring was accounted for using inverse probability of censoring weighting (IPCW). The 95% CIs and *P* values for NRI and IDI were estimated using 1,000 bootstrap resamples. For Kaplan–Meier analysis, patients were dichotomized according to the cohort median values of sST2 and GLS to avoid selecting survival thresholds based on outcome-driven optimization. ROC analysis was performed separately to evaluate discrimination and to identify the optimal cutoff using the Youden index. All *P* values were derived from two-sided tests, with results deemed statistically significant at *P* < 0.05.

## Results

### Study participants

A total of 97 patients of CMR confirmed MINOCA were included in this study. Among them, during a median follow-up of 24.2 (15.9, 36.6) months, 33 (34.0%) patients experienced MACE. The individual components of MACE included 5 all-cause deaths (5.2%), 7 recurrent myocardial infarctions (7.2%), 2 strokes (2.1%), 5 heart failure events (5.2%), 3 atrial fibrillation events (3.1%), and 11 angina pectoris events (11.3%) (Supplementary Table S1). Significant differences were observed between the two groups in sST2 (41.63 ± 33.55 vs. 76.10 ± 50.86 ng/mL, *P* < .001), LVEF, LGE%, LV-ESVi, and GLS (all *P* < 0.05) (Table 1).

**Table 1:** Baseline characteristics.

Variables	Total (*n* = 97)	Non-MACE (*n* = 64)	MACE (*n* = 33)	*P*
sST2, ng/mL	53.35 ± 43.25	41.63 ± 33.55	76.10 ± 50.86	<.001
LVEF, %	55.44 ± 9.44	57.04 ± 9.23	52.34 ± 9.17	0.019
LV mass, g	114.55 ± 33.34	111.70 ± 27.74	120.07 ± 42.10	0.244
LGE, %	18.19 ± 12.17	15.54 ± 10.70	23.32 ± 13.34	0.002
LV-EDVi, mL/m^2^	76.55 ± 19.38	74.36 ± 18.36	80.80 ± 20.83	0.121
LV-ESVi, mL/m^2^	41.18 ± 14.92	38.75 ± 11.69	45.89 ± 19.07	0.025
GLS, %	17.25 ± 3.98	18.16 ± 3.85	15.48 ± 3.69	0.001
Age, years	54.51 ± 10.98	53.73 ± 11.48	56.00 ± 9.96	0.338
BMI, kg/m^2^	25.34 ± 2.72	25.67 ± 2.77	24.70 ± 2.55	0.095
SBP, mmHg	131.44 ± 16.10	133.02 ± 15.39	128.39 ± 17.22	0.182
DBP, mmHg	81.73 ± 12.31	82.84 ± 12.10	79.58 ± 12.61	0.217
Heart rate, bpm	74.49 ± 13.99	74.06 ± 14.50	75.33 ± 13.10	0.674
Triglycerides, mmol/L	1.61 ± 0.94	1.55 ± 0.64	1.74 ± 1.34	0.431
Total cholesterol, mmol/L	4.31 ± 1.00	4.28 ± 1.01	4.36 ± 1.01	0.707
LDL-C, mmol/L	2.55 ± 0.82	2.55 ± 0.82	2.55 ± 0.83	0.981
HDL-C, mmol/L	1.05 ± 0.23	1.05 ± 0.22	1.06 ± 0.25	0.767
FBG, mmol/L	6.36 ± 2.55	6.28 ± 2.50	6.52 ± 2.67	0.668
eGFR, mL/min/1.73 m^2^	110.48 ± 14.78	110.51 ± 13.78	110.43 ± 16.77	0.979
hs-TnT, ng/L	339.9 (121.0, 714.0)	333.3 (129.1, 695.7)	371.5 (121.0, 738.5)	0.738
NT-proBNP, pg/mL	592.3 (233.0, 1,153.0)	573.5 (200.1, 1,141.3)	643.7 (300.5, 1,199.0)	0.351
hs-CRP, mg/L	4.40 (1.70, 14.60)	4.15 (1.20, 14.38)	8.50 (2.60, 17.60)	0.062
MVO, *n* (%)	6 (6.19)	3 (4.69)	3 (9.09)	0.683
Male, *n* (%)	46 (47.42)	29 (45.31)	17 (51.52)	0.562
Smoking, *n* (%)	44 (45.36)	27 (42.19)	17 (51.52)	0.382
Hypertension, *n* (%)	44 (45.36)	32 (50.00)	12 (36.36)	0.201
Diabetes, *n* (%)	14 (14.43)	6 (9.38)	8 (24.24)	0.095
Stroke, *n* (%)	12 (12.37)	8 (12.50)	4 (12.12)	1.000
P_2_Y_2_, *n* (%)	57 (58.76)	41 (64.06)	16 (48.48)	0.140
Aspirin, *n* (%)	93 (95.88)	62 (96.88)	31 (93.94)	0.881
Statins, *n* (%)	88 (90.72)	59 (92.19)	29 (87.88)	0.746
ACEI/ARB, *n* (%)	70 (72.16)	49 (76.56)	21 (63.64)	0.178
*β*-blockers, *n* (%)	70 (72.16)	48 (75.00)	22 (66.67)	0.386

BMI, body mass index; eGFR, estimated glomerular filtration rate; LVEF, left ventricular ejection fraction; GLS, global longitudinal strain; FBG, fasting blood glucose; LV, left ventricular; EDVi, end-diastolic volume index; LGE, late gadolinium enhanced; ESVi, end-systolic volume index; SBP, systolic blood pressure; DBP, diastolic blood pressure; ACEI, angiotension converting enzyme inhibitors; ARB, angiotensin II receptor blocker; HDL-C, high-density leptin cholesterol; LDL-C, low-density leptin cholesterol; hs-CRP, high sensitivity C-reactive protein; hs-TnT, high sensitivity troponin T; NT-proBNP, N-terminal pro-B-type natriuretic peptide; sST2, Soluble suppression of tumorigenicity 2.

### Relationship between MACE and sST2 in MINOCA patients

Cox regression analysis was used to explore the variables associated with MACE. Univariate analysis identified LGE, sST2, GLS, and LVEF associated with MACE (Table 2). Adjusting all variables with a *P* < 0.1 in univariate analysis, the results indicated that sST2 (HR 1.010, 95% CI 1.003–1.017, *p* = 0.005) and GLS (HR 0.864, 95% CI 0.780–0.956, *p* = 0.005) were independently associated with MACE (Table 2). The proportional hazards assumption was satisfied for the final Cox model, with no significant violations for sST2 (*P* = 0.895) or GLS (*P* = 0.309), and the global Schoenfeld residual test was also nonsignificant (*P* = 0.583).

**Table 2:** Association of patient characteristics with MACE: univariate and cox multivariate regression analysis.

	Univariate		Multivariate	
Variables	HR (95%CI)	P	HR (95%CI)	P
sST2, ng/mL	1.012 (1.005–1.019)	<0.001	1.010 (1.003–1.017)	0.005
LVEF, %	0.961 (0.930–0.994)	0.019		
LV mass, g	1.006 (0.997–1.015)	0.209		
LV-EDVi, mL/m^2^	1.010 (0.994–1.027)	0.231		
LV-ESVi, mL/m^2^	1.018 (0.998–1.038)	0.074		
GLS, %	0.845 (0.765–0.934)	<0.001	0.864 (0.780–0.956)	0.005
Age, years	1.004 (0.974–1.036)	0.775		
BMI, kg/m^2^	0.895 (0.782–1.026)	0.111		
SBP, mmHg	0.980 (0.959–1.001)	0.063		
DBP, mmHg	0.979 (0.952–1.007)	0.145		
Heart rate, bpm	1.006 (0.982–1.030)	0.642		
hs-TnT, ng/L	1.025 (0.761–1.381)	0.872		
NT-proBNP, pg/mL	1.097 (0.790–1.524)	0.579		
hs-CRP, mg/L	1.004 (0.992–1.017)	0.489		
Triglycerides, mmol/L	1.182 (0.860–1.626)	0.303		
Total cholesterol, mmol/L	0.942 (0.657–1.349)	0.744		
LDL-C, mmol/L	0.842 (0.539–1.314)	0.449		
HDL-C, mmol/L	1.291 (0.289–5.762)	0.738		
FBG, mmol/L	1.033 (0.916–1.164)	0.599		
eGFR, mL/min/1.73 m^2^	0.998 (0.975–1.021)	0.835		
MVO, %	1.359 (0.413–4.468)	0.614		
LGE, %	1.031 (1.006–1.058)	0.015		
Male, *n* (%)	1.160 (0.584–2.302)	0.672		
Smoking, *n* (%)	1.296 (0.653–2.573)	0.458		
Hypertension, *n* (%)	0.628 (0.309–1.277)	0.199		
Diabetes, *n* (%)	2.115 (0.953–4.695)	0.066		
Stroke, *n* (%)	0.851 (0.298–2.426)	0.763		
P_2_Y_12_, *n* (%)	0.769 (0.386–1.531)	0.454		
Aspirin, *n* (%)	0.841 (0.201–3.527)	0.813		
Statins, *n* (%)	0.762 (0.264–2.194)	0.614		
ACEI/ARB, *n* (%)	0.621 (0.305–1.267)	0.191		
β-blockers, *n* (%)	0.628 (0.303–1.302)	0.211		

BMI, body mass index; eGFR, estimated glomerular filtration rate; LVEF, left ventricular ejection fraction; GLS, global longitudinal strain; FBG, fasting blood glucose; LV, left ventricular; EDVi, end-diastolic volume index; LGE, late gadolinium enhanced; ESVi, end-systolic volume index; SBP, systolic blood pressure; DBP, diastolic blood pressure; ACEI, angiotension converting enzyme inhibitors; ARB, angiotensin II receptor blocker; HDL-C, high-density leptin cholesterol; LDL-C, low-density leptin cholesterol; hs-CRP, high sensitivity C-reactive protein; hs-TnT, high sensitivity troponin T; NT-proBNP, N-terminal pro-B-type natriuretic peptide; sST2, Soluble suppression of tumorigenicity 2.

Given the limited number of events, two additional fixed parsimonious sensitivity models were performed. In the model including sST2, age, and GLS, sST2 remained independently associated with MACE (HR 1.010, 95% CI 1.003–1.017; *P* = 0.006), as did GLS (HR 0.864, 95% CI 0.779–0.957; *P* = 0.005). Similarly, after adjustment for age and LGE%, sST2 remained independently associated with MACE (HR 1.010, 95% CI 1.003–1.018; *P* = 0.006). Complete results are presented in Supplementary Table S2.

The association between sST2 and MACE was also consistent after adjustment for clinically relevant covariates irrespective of their univariable significance. sST2 remained independently associated with MACE in the demographic/comorbidity-adjusted model (HR 1.011, 95% CI 1.004–1.019; *P* = 0.004), the biomarker/renal function-adjusted model (HR 1.012, 95% CI 1.005–1.020; *P* < 0.001), and the discharge medication-adjusted model (HR 1.011, 95% CI 1.005–1.018; *P* = 0.001). Additional adjustment for aspirin separately yielded similar results (HR 1.013, 95% CI 1.006–1.020; *P* < 0.001). Full results are provided in Supplementary Table S3.

The dose-response relationships between sST2, GLS, and MACE are shown in Figure 2, with no evidence of significant nonlinearity (both P for nonlinearity >0.05). The risk of MACE increased with increasing sST2 levels, whereas higher absolute GLS values were associated with a lower risk of MACE.

**Figure 2:**
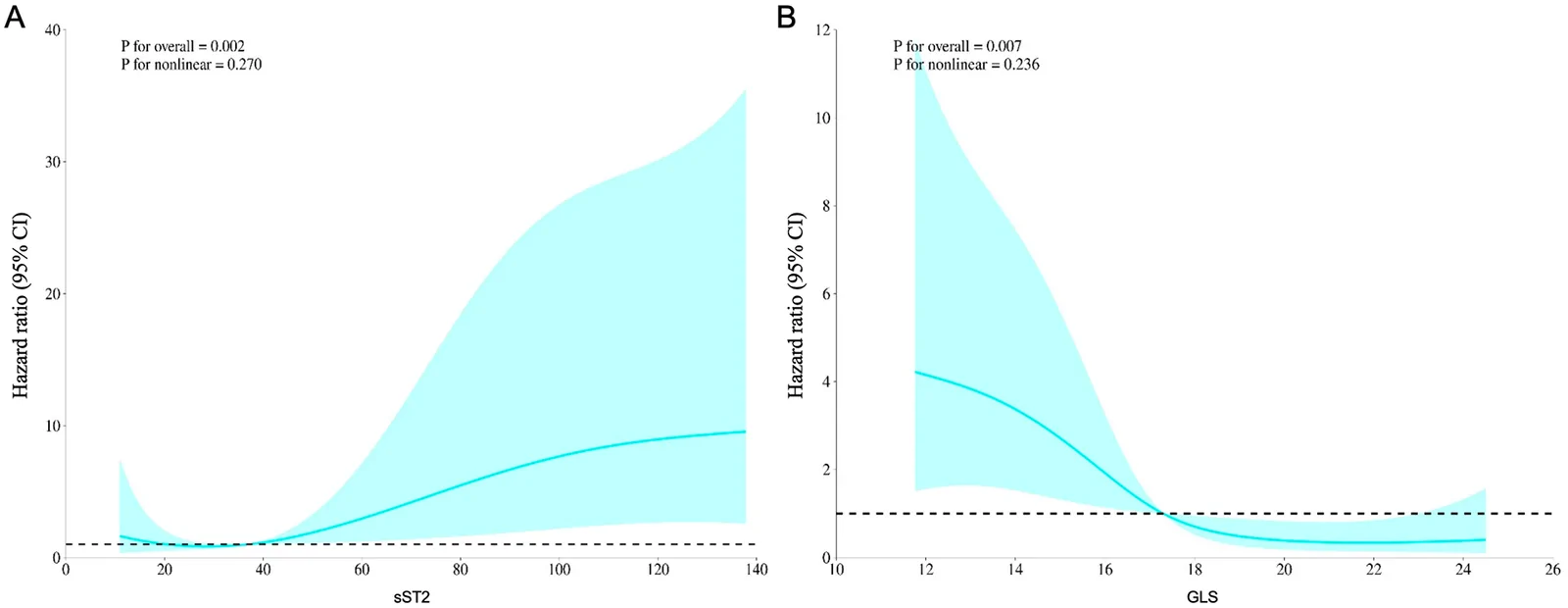
Dose-response relationship between sST2, GLS and MACE in patients with MINOCA. **(A)** Dose-response relationship between sST2 and MACE; **(B)** Dose-response relationship between GLS and MACE. sST2, soluble suppression of tumorigenicity 2; GLS, global longitudinal strain; MACE, major adverse cardiovascular events.

### Value of sST2 and GLS for identifying MACE

Conventional ROC analysis was used to explore the discrimination of GLS and sST2 for MACE. The results showed that the area under the curve (AUC) for GLS was 0.722 (95% CI 0.611–0.832, *p* < 0.001), and the AUC for sST2 was 0.696 (95% CI 0.575–0.816, *p* = 0.002) (Figure 3, Table 3).

**Figure 3:**
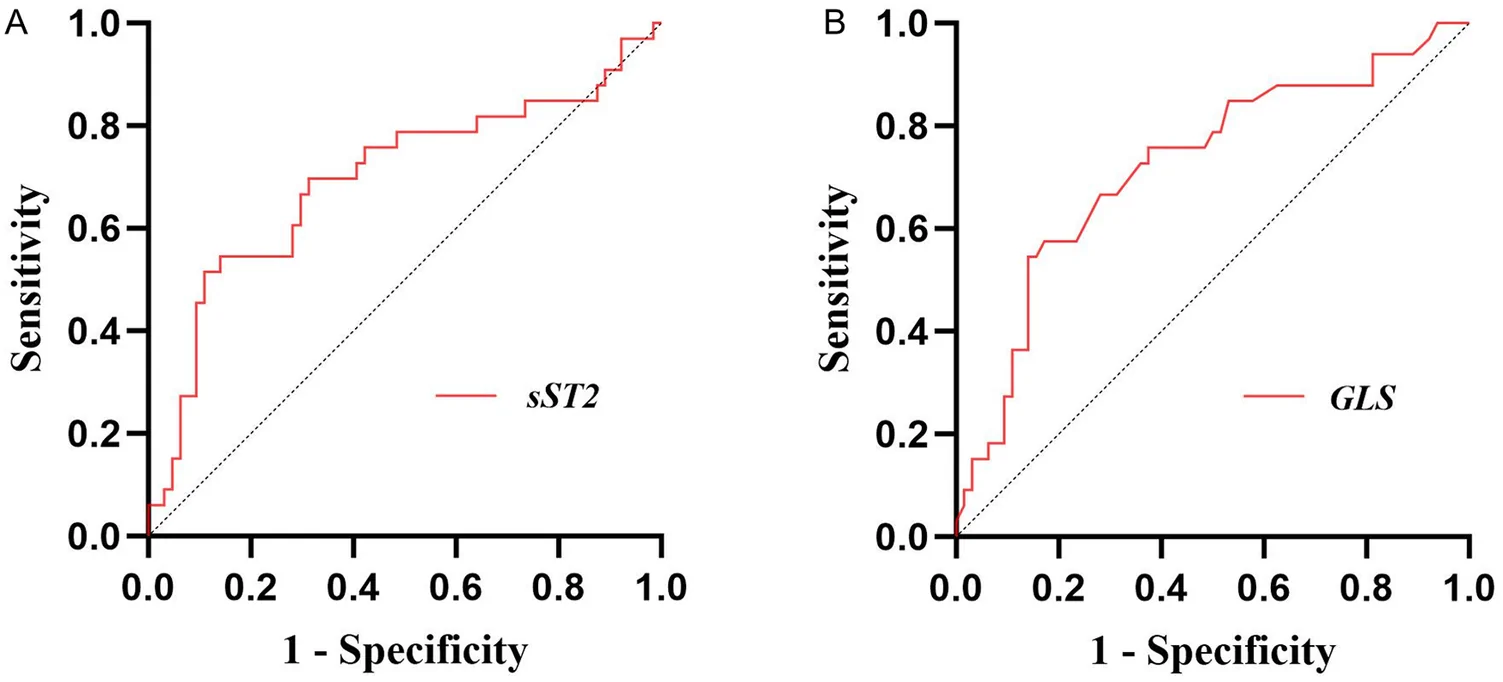
Receiver operating characteristic analysis of **(A)** sST2 and **(B)** GLS for MACE. sST2, soluble suppression of tumorigenicity 2; GLS, global longitudinal strain; MACE, major adverse cardiovascular events.

**Table 3:** ROC of GLS and sST2 for MACE.

Parameter	AUC	95%CI	P	Cutoff Value	Sensitivity (%)	Specificity (%)
sST2, ng/mL	0.696	0.575–0.816	0.002	74.53	0.515	0.891
GLS. %	0.722	0.611–0.832	<0.001	15.55	0.545	0.859

GLS, global longitudinal strain; sST2, Soluble suppression of tumorigenicity 2.

The incremental prognostic value of sST2 was further evaluated beyond conventional CMR parameters. Compared with the base Cox model including LVEF, GLS, and LGE, addition of sST2 significantly improved overall model fit in the nested Cox model comparison (likelihood ratio *χ*^2^ = 5.650, df = 1; *P* = 0.017). At the 24-month time horizon, after accounting for right censoring, addition of sST2 yielded a positive but nonsignificant continuous NRI of 0.308 (95% CI −0.247 to 0.882; *P* = 0.290) and an IDI of 0.028 (95% CI −0.009 to 0.139; *P* = 0.294) (Supplementary Table S4).

Accordingly, patients were grouped based on the median values of sST2 or GLS. The Kaplan–Meier curve demonstrated that patients with GLS ≤ 17.3% or sST2 ≥ 36.95 ng/mL had a significantly higher long-term risk of MACE compared to those with GLS > 17.3% or sST2 < 36.95 ng/mL (both log-rank *P* < 0.05) (Figure 4).

**Figure 4:**
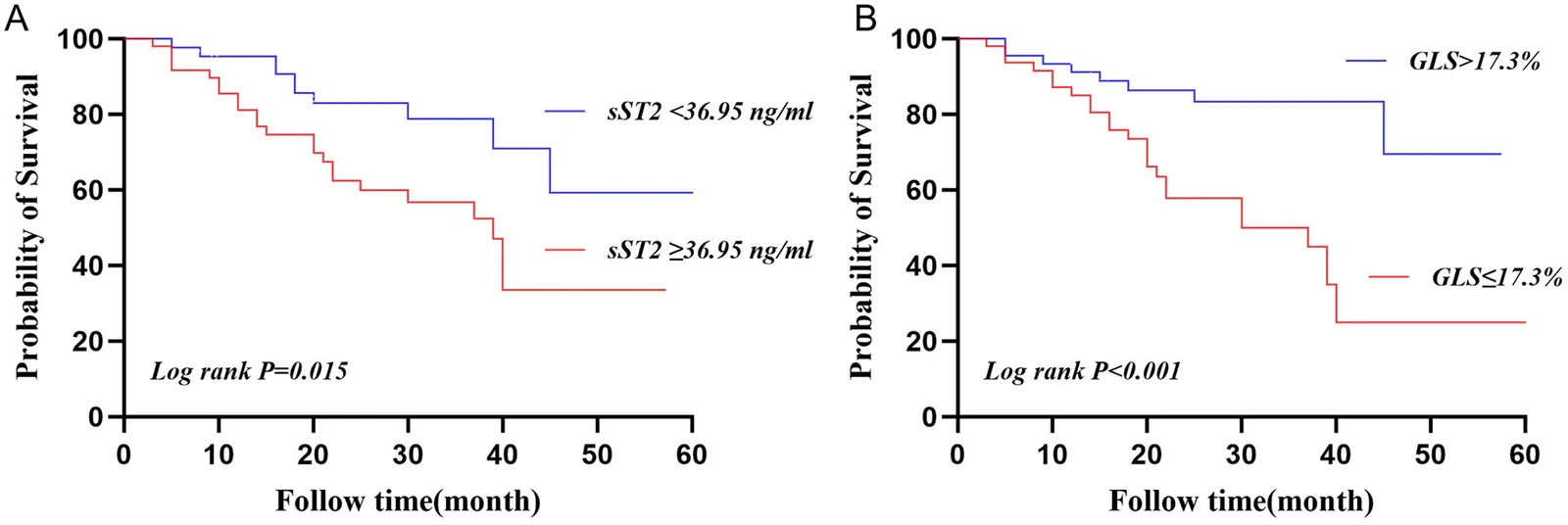
Kaplan–meier curve for patients based on the median values of sST2 or GLS. **(A)** Kaplan–Meier curve of sST2 for MACE; **(B)** Kaplan–Meier curve of GLS for MACE. GLS, global longitudinal strain; sST2, soluble suppression of tumorigenicity 2; MACE, major adverse cardiovascular events.

## Discussion

To the best of our knowledge, this study is the first to investigate the relationship between sST2 and MACE in CMR confirmed MINOCA patients. The main findings of this study are as follows: First, elevated sST2 was an independent risk factor for MACE in MINOCA patients. Second, patients with sST2 levels above the cohort median (36.95 ng/mL) had a significantly higher long-term risk of MACE.

MINOCA is associated with poor prognosis, which prompts clinicians to clarify the underlying etiology so that effective management measures can be developed (2, 3). CMR can provide etiology-specific diagnoses such as AMI, TS, myocarditis, and non-ischemic cardiomyopathy, of which the latter three would lead to the exclusion of MINOCA because they do not meet the latest definition of AMI (1, 4). In our study (median time to CMR scan was 3 days after admission), 57 (19.9%) patients were found to have a normal CMR and 229 (80.1%) patients were clarified as to the etiology. This is consistent with a previous prospective study, in the Stockholm Myocardial Infarction with Non obstructive Coronary arteries (SMINC-2), where the median time to CMR scan was also 3 days and the etiology was clarified in 77% patients (28). Of note, in previous studies, CMR has shown great variability in its ability to diagnose the etiology of MINOCA. Only 47% of patients who underwent CMR at a median of 12 days had their etiology clarified, which is due to the fact that the timing of the CMR scan is critical for the diagnosis of the etiology (28, 29). LGE and edema show significant changes over time, and a previous study demonstrated that the number of patients presenting with LGE nearly doubled and the prevalence of edema was 6 times higher in a subgroup of patients who underwent early CMR (<2 weeks) (28, 30). Therefore, early CMR in patients with MINOCA is critical (31).

Although CMR for the diagnosis of patients with MINOCA has been recommended by guidelines and consensus, risk stratification for MINOCA is still lacking. Myocardial fibrosis is one of the mechanisms central to the poor prognosis of cardiovascular disease. sST2, a marker of myocardial fibrosis, has been shown to be involved in the progression of several cardiovascular diseases and may improve risk stratification (15, 32, 33). In clinical studies, serum sST2 was elevated early after AMI and was significantly associated with poor prognosis (32, 34). Previous studies have shown prognostic associations of hs-cTnT and NT-proBNP in MINOCA, whereas evidence for fibrosis-related biomarkers such as galectin-3 remains limited. sST2 may therefore provide complementary prognostic information reflecting myocardial stress and fibrotic signaling (35, 36). A recent CMR study demonstrated that baseline levels of sST2 remained independently associated with distant LV remodeling in MI after adjusting for risk factors including MVO, infarct size (33). Our study innovatively identified elevated sST2 as an independent risk factor for MACE in patients with MINOCA. Mechanistically, sST2 is a reliable marker of myocardial fibrosis, which is a recognized risk factor for poor prognosis in patients with MI (15, 33). GLS, a traditional CMR factor for poor prognosis in MI (20, 37), was also shown to be associated with poor prognosis in patients with MINOCA in our study. Patients with lower GLS or higher sST2 had a significantly higher long-term risk of MACE. Our study suggests that CMR not only has a reliable etiologic diagnostic ability for MINOCA, but is also an important tool for risk stratification. Furthermore, addition of sST2 to conventional CMR parameters significantly improved overall model fit in the nested Cox model comparison. Although the 24-month censoring-adjusted NRI and IDI showed positive point estimates, neither reached statistical significance. Taken together, these findings suggest that sST2 may provide incremental prognostic information beyond established structural and functional CMR markers, although its incremental reclassification and discrimination performance requires confirmation in larger cohorts.

sST2, as an easily accessible marker, may be a useful tool for risk stratification of patients with MINOCA and for screening of high-risk patients. Thus, our study provides additional insight into the prognostic value of patients with MINOCA, and more rigorous follow-up and management may be required for patients with high baseline sST2 levels and or low GLS.

### Limitations

Several limitations should be noted in this study. First, this was a retrospective observational study and was therefore subject to inherent selection and information biases. Second, although rehospitalization for angina pectoris is clinically relevant, it is a softer endpoint than death, recurrent MI, or heart failure and accounted for a substantial proportion of the composite events. Third, the relatively small sample size and limited number of MACE events may increase the risk of model overfitting and reduce the precision of effect estimates. Although the final primary Cox model contained only two covariates, the events-per-candidate-variable ratio during the initial variable-selection stage was relatively low. We therefore performed two fixed parsimonious sensitivity models, each with an EPV of 11.0, and observed a consistent association between sST2 and MACE. Nevertheless, residual model optimism cannot be completely excluded, and these findings require validation in larger independent cohorts. In addition, conventional rather than time-dependent ROC analysis was used; therefore, the reported AUC values did not account for follow-up time or censoring and should be interpreted as exploratory measures of discrimination. Fourth, intracoronary imaging, particularly OCT, was not routinely performed in our cohort. Although CMR enabled the exclusion of non-ischemic mimickers and confirmation of an ischemic myocardial injury pattern, the absence of intracoronary imaging may have limited further identification of underlying coronary mechanisms, such as plaque disruption or other subtle coronary abnormalities. Given the increasing role of intracoronary imaging in contemporary MINOCA evaluation, future studies integrating CMR with OCT or other intracoronary imaging techniques are warranted to provide more comprehensive etiologic characterization (38). Fifth, more basic studies may be needed to elucidate the specific mechanism of action regarding the poor prognosis and sST2. However, there is sufficient evidence in our study to suggest an association between sST2 and MACE in patients with MINOCA.

## Conclusions

Elevated sST2 is independently associated with adverse outcomes in patients with CMR-confirmed MINOCA. sST2 may serve as a practical biomarker for risk stratification in this population.

## Data Availability

The raw data supporting the conclusions of this article will be made available by the authors, without undue reservation.
