# Supplementary material for: Prognostic value of soluble ST2 in cardiac magnetic resonance defined myocardial infarction with non-obstructive coronary arteries
Source: Front Cardiovasc Med. 2026 Sep 15;13:1917954. doi: 10.3389/fcvm.2026.1917954 (PMC13619514; doi:10.3389/fcvm.2026.1917954)
Supplement: Supplementary file 1 [file Datasheet1.docx]

**Table S1. Adverse Events During Follow-up in Patients with “True MINOCA”**

| **Adverse events, n (%)** | **All patients** |
| --- | --- |
| MACE | 33 (34.0) |
| All-cause death | 5 (5.2) |
| Recurrent myocardial infarction | 7 (7.2) |
| Stroke | 2 (2.1) |
| Heart failure | 5 (5.2) |
| Atrial fibrillation | 3 (3.1) |
| Angina pectoris | 11 (11.3) |

MACE, major adverse cardiovascular events; MINOCA, myocardial infarction with non-obstructive coronary arteries.

**Supplementary Table S2. Parsimonious sensitivity Cox regression analyses for MACE**

| **Variable** | **Model 1 HR (95% CI)** | **P value** | **Model 2 HR (95% CI)** | **P value** |
| --- | --- | --- | --- | --- |
| sST2, ng/mL | **1.010 (1.003–1.017)** | **0.006** | **1.010 (1.003–1.018)** | **0.006** |
| Age, years | 1.000 (0.967–1.033) | 0.991 | 0.995 (0.963–1.029) | 0.781 |
| GLS, % | **0.864 (0.779–0.957)** | **0.005** | — | — |
| LGE, % | — | — | 1.020 (0.994–1.046) | 0.142 |

Model 1 included sST2, age, and GLS; Model 2 included sST2, age, and LGE%. All covariates were entered simultaneously without stepwise variable selection. Each model included three covariates for 33 MACE events, corresponding to an events-per-variable ratio of 11.0. CI, confidence interval; GLS, global longitudinal strain; LGE, late gadolinium enhancement; MACE, major adverse cardiovascular events; sST2, soluble suppression of tumorigenicity 2.

**Supplementary Table S3. Sensitivity Cox regression analyses adjusted for clinically relevant confounders**

| **Variable** | **HR (95% CI)** | **P** |
| --- | --- | --- |
| Model 1. Demographic/comorbidity-adjusted model | | |
| sST2, ng/mL | 1.011 (1.004–1.019) | 0.004 |
| Age, years | 0.993 (0.960–1.027) | 0.670 |
| Male | 1.124 (0.553–2.285) | 0.747 |
| Diabetes | 2.036 (0.869–4.767) | 0.102 |
| Hypertension | 0.796 (0.366–1.734) | 0.566 |
| Model 2. Biomarker/renal function-adjusted model | | |
| sST2, ng/mL | 1.012 (1.005–1.020) | <0.001 |
| NT-proBNP | 1.001 (0.724–1.385) | 0.994 |
| hs-TnT | 1.073 (0.798–1.442) | 0.642 |
| eGFR | 1.003 (0.980–1.026) | 0.813 |
| Model 3. Discharge medication-adjusted model | | |
| sST2, ng/mL | 1.011 (1.005–1.018) | 0.001 |
| P2Y12 inhibitor | 0.901 (0.424–1.914) | 0.787 |
| Statins | 1.102 (0.316–3.838) | 0.879 |
| ACEI/ARB | 0.672 (0.314–1.436) | 0.305 |
| β-blockers | 0.725 (0.311–1.689) | 0.456 |

All covariates were entered simultaneously without stepwise variable selection. NT-proBNP and hs-TnT were natural logarithm-transformed. Aspirin was assessed separately because 93 of 97 patients (95.9%) received aspirin at discharge; adjustment for aspirin did not materially alter the association between sST2 and MACE (HR 1.013, 95% CI 1.006–1.020; P < 0.001).

**Supplementary Table S4. Incremental prognostic performance of sST2**

| Analysis | Estimate | 95% CI / df | P value |
| --- | --- | --- | --- |
| Nested Cox likelihood-ratio test | χ² = 5.650 | df = 1 | 0.017 |
| 24-month continuous NRI (>0) | 0.308 | −0.247 to 0.882 | 0.290 |
| 24-month IDI | 0.028 | −0.009 to 0.139 | 0.294 |

The base Cox model included LVEF, GLS, and LGE, whereas the expanded model additionally included sST2. The likelihood-ratio test compared the two nested Cox models. For NRI and IDI, predicted MACE risks were evaluated at 24 months, which closely approximated the median follow-up duration of 24.2 months. Right censoring was accounted for using inverse probability of censoring weighting (IPCW). The 95% CIs and P values for NRI and IDI were estimated using 1,000 bootstrap resamples. GLS, global longitudinal strain; IDI, integrated discrimination improvement; IPCW, inverse probability of censoring weighting; LGE, late gadolinium enhancement; LVEF, left ventricular ejection fraction; MACE, major adverse cardiovascular events; NRI, net reclassification improvement; sST2, soluble suppression of tumorigenicity 2.
